# A symposium of seven sages of the world in cardiovascular surgery; the revival of the ancient symposium

**DOI:** 10.1186/1749-8090-8-S1-O162

**Published:** 2013-09-11

**Authors:** C Lolas

**Affiliations:** 1 University of Athens, "Ygeia" Hospital, Athens, Greece

## 

The wisdom of the Seven Sages of Antiquity, who were philosophers and scientists, played a catalytic role in the exchange of ideas and in bringing the people of different Greek city states together in the process of forming an organized society. In today’s world, facing the side effects of globalization (commercial exploitation, the influence of the internet and mass media, immigration, etc.) we should revive the ancient Symposium of the Seven Sages seeking to share the experience and the wisdom of the Sages of the modern world of all Arts and Sciences for guidance in solving the immense challenges of the third millennium.

The theme of the cardiovascular surgery for the first Symposium was chosen because the heart symbolizes love and life, from time it first begins beating inside the mother’s womb, until it finally ceases to beat at death. Seven legends of cardiovascular surgery of the world were invited to participate in the symposium. They were innovators and great teachers who paved the way for the next generation of Cardiac Surgeons, enabling them to continue their efforts to improve and prolong life, and relieve pain and suffering. The Seven Sages, who participated in the first modern symposium, were, in alphabetical order: Alain Carpentier (France), Denton Colley (USA), Adib Jatene (Brazil), Donald Ross (UK), Albert Starr (USA), Juro Wada (Japan) and Magdi Yacoub (UK).

In two round table panel discussions which took place in Athens and in Delphi on May 6 & 7, 2007, the seven wise men answered questions regarding general and medical issues like the environmental research, war, space exploration, to name a few, as well as answering questions related to Cardiovascular Surgery.

The purpose of this communication is to present this unique event, particularly to young colleagues, giving an opportunity to share the wisdom of these seven legends of our specialty in a special forum. Secondly, the purpose is to revive the Symposium of the Seven Sages of Antiquity in our modern era, creating a forum by which to examine the wisdom of other individuals in all the Arts and Sciences.

